# Fuzzy Logic-Based and Nondestructive Concrete Strength Evaluation Using Modified Carbon Nanotubes as a Hybrid PZT–CNT Sensor

**DOI:** 10.3390/ma14112953

**Published:** 2021-05-30

**Authors:** Najeebullah Tareen, Junkyeong Kim, Won-Kyu Kim, Seunghee Park

**Affiliations:** 1 Department of Civil, Architectural & Environmental System Engineering, Sungkyunkwan University, 2066 Seobu-ro, Suwon-si 16419, Korea; engr.najeeb12@gmail.com; 2 Safety Inspection for Infrastructure Laboratory (SIIL), Advanced Institute of Convergence Technology (AICT), Suwon-si 16229, Korea; junk135@nate.com; 3 Department of Convergence Engineering for Future City, Sungkyunkwan University, 2066 Seobu-ro, Suwon-si 16419, Korea; kwk1104@gmail.com; 4 School of Civil, Architectural Engineering and Landscape Architecture, Sungkyunkwan University, 2066 Seobu-ro, Suwon-si 16419, Korea

**Keywords:** carbon nanotube (CNT) sensors, smart materials, nondestructive testing (NDT), electromechanical impedance (EMI), maturity method, early-age concrete strength, piezoelectric sensors, structural health monitoring (SHM)

## Abstract

Concrete strength and factors affecting its development during early concrete curing are important research topics. Avoiding uncertain incidents during construction and in service life of structures requires an appropriate monitoring system. Therefore, numerous techniques are used to monitor the health of a structure. This paper presents a nondestructive testing technique for monitoring the strength development of concrete at early curing ages. Dispersed carbon nanotubes (CNTs) were used with cementitious materials and piezoelectric (PZT) material, a PZT ceramic, owing to their properties of intra electromechanical effects and sensitivity to measure the electromechanical impedance (EMI) signatures and relevant properties related to concrete strength, such as the elastic modulus, displacement, acceleration, strength, and loading effects. Concrete compressive strength, hydration temperature, mixture ratio, and variation in data obtained from the impedance signatures using fuzzy logic were utilized in the comparative result prediction method for concrete strength. These results were calculated using a fuzzy logic-based model considering the maturity method, universal testing machine (UTM) data, and analyzed EMI data. In the study, for data acquisition, a hybrid PZT–CNT sensor and a temperature sensor (Smart Rock) were embedded in the concrete to obtain the hydration temperature history to utilize the concrete maturity method and provide data on the EMI signatures. The dynamic changes in the medium caused during the phase in the concrete strengthening process were analyzed to predict the strength development process of concrete at early curing ages. Because different parameters are considered while calculating the concrete strength, which is related to its mechanical properties, the proposed novel method considers that changes in the boundary condition occurring in the concrete paste modify the resonant frequency response of the structure. Thus, relating and analyzing this feature can help predict the concrete strength. A comprehensive comparison of the results calculated using the proposed module, maturity method, and cylindrical specimens tested using the UTM proved that it is a cost-effective and fast technique to estimate concrete strength to ensure a safe construction of reinforced cement concrete infrastructures.

## 1. Introduction

To improve the construction quality and provide a safe environment for the construction of infrastructure, numerous techniques have been developed to monitor the health of a structure. To conduct such monitoring during construction and the service life of infrastructures, the tests must be advantageous and of high accuracy. Several in situ tests have been conducted on concrete structures [1,2,3,4,5,6,7,8,9,10,11]. These tests can be destructive or nondestructive testing (NDT) types. NDT is preferred because real structural elements are not damaged, and structural members are not displaced. Moreover, these tests yield a large amount of data at a relatively low cost. Some of the commonly used NDT techniques are Schmidt rebound hammer tests, acoustic testing, resonant frequency testing, radiography, testing using an isothermal calorimeter, ultrasonic pulse velocity tests, and the maturity method [12,13,14,15,16,17,18,19,20,21,22]. Concrete is a heterogeneous mixture of materials such as cement, water, coarse aggregates, fine aggregates, and some admixtures. Therefore, to ensure the durability and proper strength gaining by structural members, it is important to determine the properties of concrete paste at various curing ages. The early-age properties of concrete play a significant role in the structural safety of structures. The concrete strength must be monitored from the 1st day to the 28th day of curing and can also be monitored subsequently. For some large concrete structures, such as high-rise reinforced cement concrete buildings, bridges, and dams, continuous monitoring of their structural members or the overall structures at the early-construction and functional stages is highly challenging, technical, and time-consuming owing to their massive sizes. The conventional techniques, such as visual inspection by trained professionals, cannot provide interpretive health assessment and are unsuitable for such structures. For the structural integrity and safety maintenance of massive structures, they must be equipped with advanced instruments, and appropriate techniques for structural health monitoring must be applied. Thus, there is a need to develop automated systems for the constant health monitoring and structural damage detection of concrete structures. To meet these requirements, we examine the combined use of smart materials of carbon nanotubes (CNTs) and piezoelectric (PZT) materials as a modified sensor, owing to their electrotechnical effects, to specify the early-age concrete strength based on the physical response properties of a concrete structure.

CNTs are a focus in global research because of their excellent properties of electrical network formation and piezoresistivity. Since Iijima introduced CNTs in 1991 [23], they have been extensively used for achieving various applications. CNTs have remarkable properties and interesting applications related to their strength (Young’s modulus of approximately 1.8 TPa), metallic properties, and high aspect ratio of >500. Because of their high aspect ratio, CNTs can easily react and serve as conductive agents and provide a reinforcement network when the CNT content is as low as 0.1% [24,25,26,27]. Owing to their Young’s modulus being higher than that of cement, CNTs require a greater tensile force for their elongation. Hence, CNTs can act as reinforcements in specimens; they can also form composites because of their structural homogeneity [28]. CNTs can be single-walled nanotubes (SWNTs) or comprise many shells as in multi-walled nanotubes (MWNTs). CNTs are formed from single sheets of graphite as seamless tubular structures with lengths and diameters in the ranges of 0.2–5 μm and 1–20 nm, respectively. In most cases, the bulk forms of CNTs, such as powders, papers, films, and aligned stacks, cannot be used. Owing to the microscopic form of an individual CNT, inherent properties present poor translation. Therefore, CNTs are generally combined with other materials to form hybrid materials, alloys, or composites [29]. Some studies have investigated the use of CNTs as fillers in various polymers, such as conjugated polymers, elastomers, or thermosets, to form composites. Our concept of adding PZT pitches to CNTs is relevant for introducing a novel sensor for measuring the dynamic effects of concrete composites. For structural health monitoring and measurement, the dynamic changes occurring in concrete structures can be measured based on toughness, strength, and other properties. In this study, the stress and strain change considerably with the variation in the electrical properties of the CNTs. The host structure presents a linear and reversible piezoresistive response, which was also studied by Dharap and Grow [30,31,32]. Various studies on CNTs are related to the use of nanotube membranes. Recently, CNTs have been studied extensively for their sensing properties; in composites, CNTs are sensitive to dynamic changes in the structures. CNTs can not only form a good bond with cement mortar, but also serve as sensors for detecting mechanical changes in structures. Studies have been conducted on the use of CNTs as good sensing materials for measuring dynamic effects [33,34,35,36,37,38,39,40,41,42].

## 2. Methodology

### 2.1. Use of CNTs as Sensors

**Figure 1:**
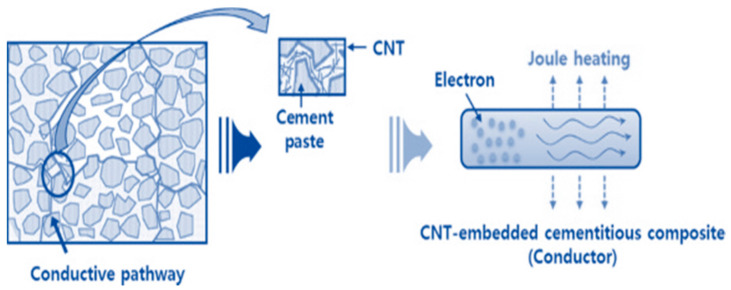
Signal processing pathway of carbon nanotube (CNT) specimens.

Cementitious materials are quasi-brittle and present electrical and thermal insulation features. Thus, their sensitivity is affected by the addition of nanofibers, which inverts the cementitious matrices, thereby modifying the properties of the concrete. The signal processing pathway is shown in Figure 1.

Research and development in the field of structural health monitoring has generated significant interest in nanosized fibers, such as CNTs, for modifying the properties of concrete. The metallic property of piezoresistivity of CNTs enables them to detect the changes in the stress/strain in a specimen. Furthermore, CNTs can simultaneously act as reinforcement elements for improving the toughness of concrete. This study demonstrates that the sensitivity of CNTs to strain is enhanced when PZT pitches are added, which can be optimized by the remarkable maintenance of the intra-mechanical and electrical properties of PZT materials and CNTs. For such materials, the stress variations are correlated to the variations in their properties, such as impedance and electrical resistance. These specimens actuate to generate vibrations and actuation in a concrete structure and, subsequently, act as a sensor to detect the vibrations and waves for the detection of the dynamic response of the host structure.

### 2.2. Fabrication of CNT Specimens

The fabrication process of the proposed smart hybrid PZT–CNT materials involved two steps.

**Figure 2:**
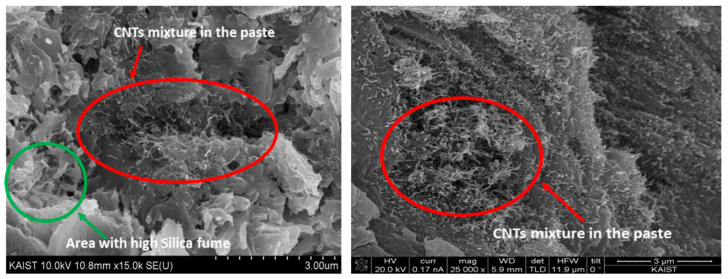
SEM images for the CNT dispersion in the cementitious paste.

The first step was the synthesis of the CNT specimens, which was conducted using a simple process following the American Society for Testing and Materials (ASTM) C 305. A standard Hobart mixer was used for the preparation of the paste. As the cementitious materials, Portland cements containing various concentrations of iron oxide particles (Fe_2_O_3_) and a sulfate-resistant material (4 wt.%) were used. For the good growth of CNTs, Al_2_O_3_, SiO_2_, and MgO are effective mixing materials; therefore, they were employed as is done typically. To ensure cost effectiveness and a low decomposition temperature, acetylene was used as the main source of carbon. According to Kim, the dispersion of CNTs can be enhanced by minimizing the water content, which increases the collision probability between particles of silica fume and the agglomerated CNTs in the matrix of a CNT–cement composite. Some details about dispersion of CNT partials in the prepared paste are given in Figure 2, which has been scanned by electron microscope and was taken for the same ratio paste by Kim [43]. In the photos, CNT bonds’ connectivity can be seen, which provides the main features for the electrical and thermal properties of the specimens.

**Figure 3:**
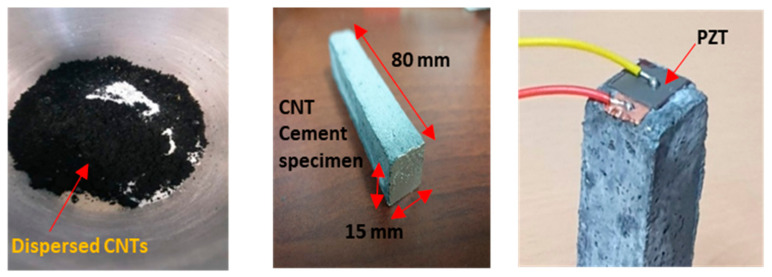
CNT–cement and CNT–piezoelectric (PZT) specimens.

In total, 16 CNT–cement paste specimens that had dimensions of 15 × 15 × 80 mm^3^ were prepared to serve as sensors. Here, “0.22%” CNTs were added, by weight, to the weight of cement; moreover, to enhance the effects of silica fume to the CNT–cement composites, 18% was added by weight to the cement. The amount of water in the composite was minimized to 28% by weight. In terms of the cement weight ratio, 100% aggregates and 1.5% admixture were added to the composite. Some of the specimens are shown in Figure 3.

**Figure 4:**
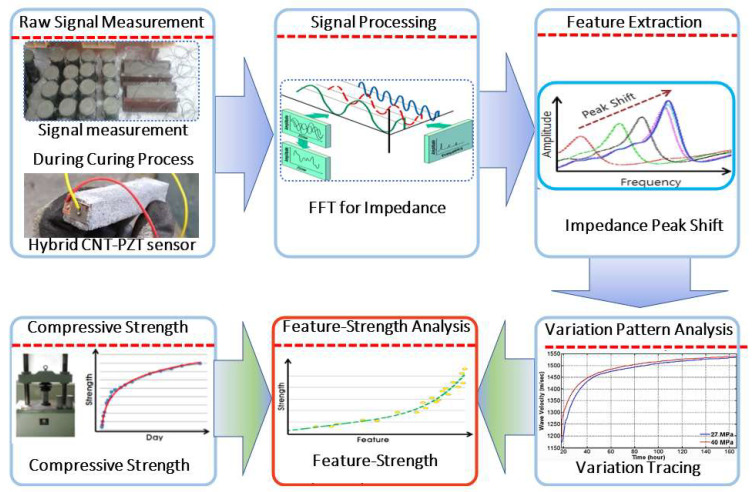
Data acquisition and analysis process.

The cement, CNTs, silica fume, and aggregates were mixed in dried forms for 5 min to ensure good dispersion of the CNTs. Subsequently, the admixture and water were added, and the mixture was mixed for 3 min. Following this, the composite paste was poured into a plastic mold with the aforementioned dimensions, and each specimen was cured at a temperature of 23 ℃. Per the specifications of ASTM C 39, all specimens were tested for compressive strength and porosity. According to some previous studies, the total porosity of a concrete specimen can be obtained using a water absorption method known as mercury intrusion porosimetry [43]. Figure 4 presents a flowchart of the measurement process used in this study.

**Figure 5:**
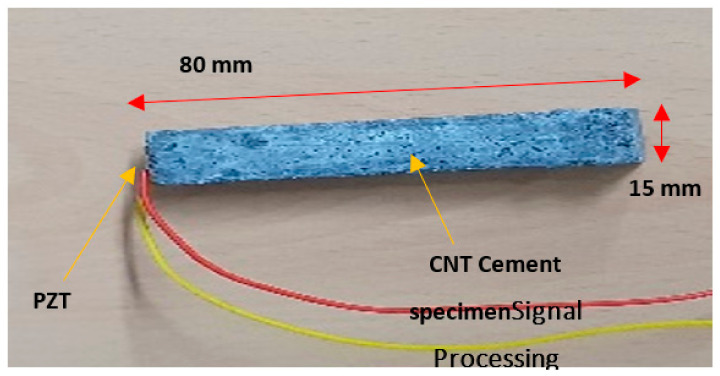
Modified PZT–CNT sensor with dimensional details.

In the second step, following the completion of the curing process for the CNT–cement paste specimens, a 10 × 10 mm^2^ PZT ceramic was attached, as shown in Figure 5. For the PZT materials, the American piezoceramics (APC) Materials 850 WEB Series (Mackeyville, PA, USA) was used, whose specifications are listed in Table 1. The specimens were also tested for resistance and phase changes to determine the polarization.

### 2.3. PZT Sensor

PZT materials can interconvert their electrical and mechanical energies; therefore, the addition of PZT material enhances the polarization of the electrical signal response [44,45]. This property of PZT materials can be used to enable them to act as a sensor and an actuator for detecting electromechanical impedance (EMI) signature changes, wave propagation, and vibrations [1,6,46,47,48]. Different techniques that employ PZT materials as actuators and sensors to measure the dynamic changes in concrete structures and related parameters can be utilized for estimating the concrete strength. From the dynamic change in the structure, the variations in the EMI, in turn, can be obtained [19].

Because the PZT material exhibits a linear electrical behavior,

(1) D=εE,

$$D_{i}=\varepsilon_{ij}E_{i}.$$

In Equation (1), *E* is the strength of the electric field, ε is the free-body permittivity, and *D* is the displacement of the electric charge density. The dielectric constant of the medium is a proportionality factor related to the strength of the electric field and the electric displacement. Therefore, Equation (1) represents the electric charge density’s tensor of displacement.

From Hook’s law,

(2) S=sT,

$$S_{ij}=S_{ij}T_{j}.$$

Combining Equations (1) and (2) yields

(3) $$S=s^{E}T+dE,$$

$$D=dT+\varepsilon^{T}E.$$

Here, *S* is the strain, $\varepsilon^{T}$ is the dielectric constant of the PZT material under a constant stress, *T* is the stress, and $s^{E}$ is the constant electrical field compliance.

The data of the sensor directly depend on the parameters related to the concrete strength, such as the stiffness, curing time, temperature, modules of elasticity, hydration temperature, and humidity. Therefore, for different mixtures, the conditions were varied, and the water–cement ratio was changed while keeping the concrete type constant. To obtain the design strength, appropriate curing of the specimen and maintenance of the water–cement ratio are required. For this purpose, Abrams’ equation can be considered.

(4) fC=k1k2wC.

Here, wC is the water–cement ratio and $k_{1}$,$k_{2}$ are the empirical constants.

Concrete strength decreases with increasing water and air contents, whereas it increases with the addition of cement paste. The temperature and humidity directly affect the concrete strength at the early curing ages after the concrete paste is poured. A long curing time with appropriate moisture results in a high strength for a concrete mixture at a specified water–cement ratio. The American Concrete Institute Committee 209 (2008) recommends the following strength relationship for a concrete containing normal Portland cement, which has a high moisture content and is cured well [49]:

(5) $$f_{cm}\left(t\right)\;=f_{c_{28}}\left[\frac{t}{4+0.85t}\right].$$

In Equation (5), $f_{cm}\left(t\right)$ denotes the mean compressive strength at an age of *t* days and $f_{c28}$ represents the strength on the 28th day. Similarly, the Comité Européen du Béton-Fédération Internationale du Béton models suggest the following compressive strength and time relationship shown in Equation (6).

(6) $$f_{cm}\left(t\right)\;=\exp\left[S\left(1-\sqrt{\frac{28}{t/t_{1}}}\right)\right]f_{cm}.$$

Here, $f_{cm}\left(t\right)$ is the mean compressive strength at an age of *t* days and $f_{cm}$ represents the strength on the 28th day; the strength on the 1st day is $t_{1}$. In the Equation, *s* is a cement-type dependent coefficient, i.e., for quick-strength cement, cement with normal hardening, and cement with slow hardening, its values are 0.20, 0.25, and 0.38, respectively [50]. For a concrete with a long moisture time that has been cured in a high-temperature environment, the strength development is rapid [47].

### 2.4. Casting the Hybrid PZT–CNT Sensor in Concrete

Addition of CNTs to the cementitious material increases the heat and mechanical response effects in the paste. The dynamic response obtained from the electromechanical and ultrasonic waves yields the boundary changes in the material, from which the phase variations of the concrete paste at early curing ages can be estimated. A noise-free data procurement procedure is introduced to extract the features for the prediction of the concrete compressive strength. The concrete strength estimation technique uses algorithms based on fuzzy logic for the extraction of the feature variation in the host structure.

**Figure 6:**
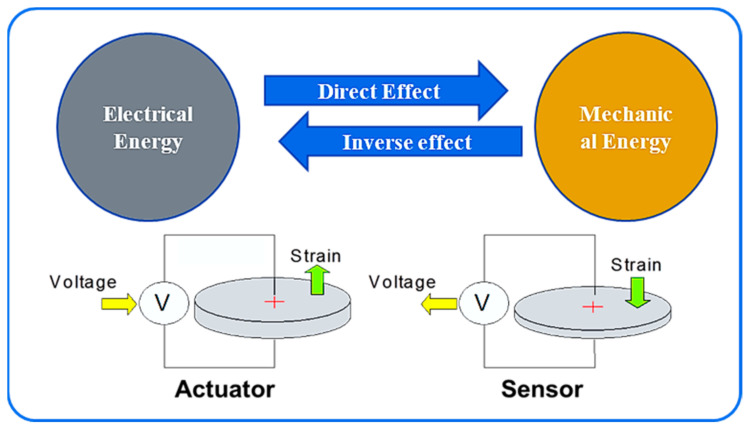
Schematic of PZT–CNT sensor energy interconversion process.

The transducer was polarized for the measurement of the features from the signal processing of the EMI. To detect the effects, pattern changes in concrete, signal processing, and dynamic responses of the structure, we used PZT–CNT sensors (APC international, Mackeyville, PA, USA) to intensify the sensibility of the transducer. As discussed earlier, the PZT material can generate a signal to actuate a specimen and measure the propagated response signal based on piezoelectricity, whereas CNTs can respond to the stresses developed by the PZT actuators in the specimen. An example of the energy interconversion is presented in Figure 6.

## 3. Experimental Setup

### 3.1. Data Acquisition Process

**Figure 7:**
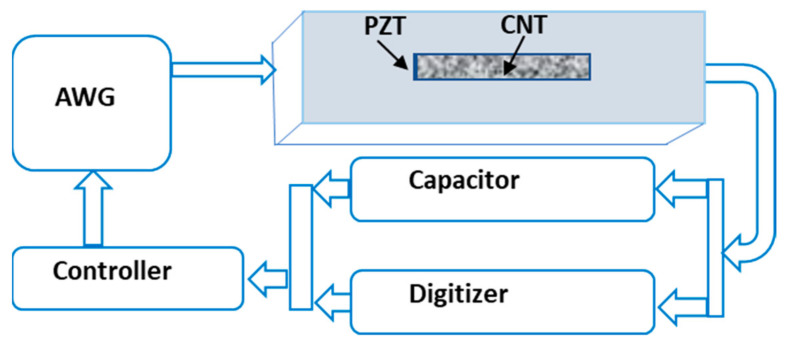
Schematic of the embedded PZT–CNT sensor and data acquisition process.

**Figure 8:**
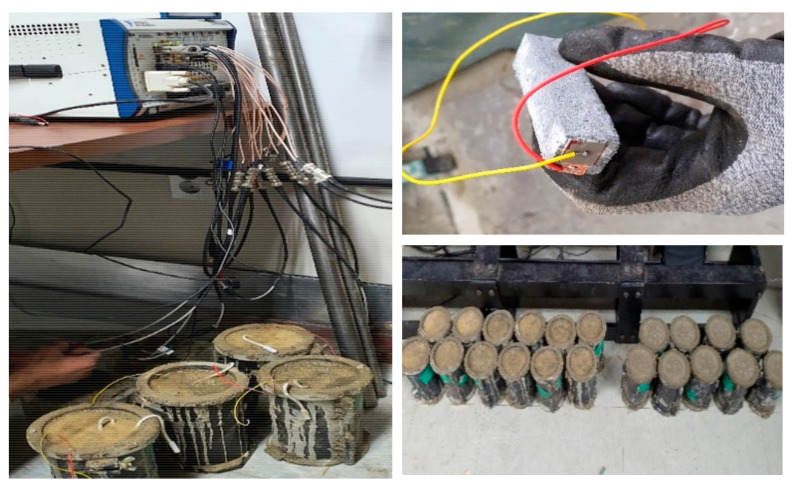
PZT–CNT sensor casting and data acquisition process.

To estimate the concrete compressive strength, we have used the sensor data collected from the early pouring time till the 28th day of curing. We used the EMI as a function of the curing parameter and the calculated cross-correlations (1-CC) among the data. As the curing time progresses in the initial sitting time, the concrete paste phase changes with the increasing stiffness of the material. The dynamic change responses of the structure were monitored by the hybrid PZT–CNT sensor, and the EMI data depended on the changes in the frequency and time. A fuzzy logic-based module was used to analyze and simplify the data precisely with a time factor and yield the EMI in the signals of the structure, which were further used to predict the compressive strength. The algorithm of the fuzzy logic-based module was implemented in MATLAB by employing several parameters and quantifying rules to categorize and simulate the data for strength evaluation. The properties of the specimens were continuously measured in 30 min intervals by the hybrid PZT–CNT sensors, using a signal digitizer computer setup. The EMI was measured as a function of the curing time. The data acquisition process is presented in Figure 7. For data measurement of the specimens, a signal digitizer, multiplexers, an arbitrary waveform generator, and a controller were assembled to control the data processing, as shown in Figure 8.

### 3.2. Concrete Mixtures

The hybrid PZT–CNT sensors were designed for the measurement of ordinary concrete strength. Two types of concrete mixtures were prepared for the experiment, whose details are provided in Table 2.

For data acquisition, the experimental setup was arranged before casting the concrete. The experiment was conducted at the room temperature of 23 °C because of the use of ordinary Portland cement concrete. Two types of concrete mixtures, with designed compressive strengths of 18 MPa and 20 MPa, respectively, were prepared. For the data acquisition, 25 cylindrical specimens of each type of concrete mixture were casted, into which the sensors were embedded, and the remaining specimens were cured for the compressive strength test by a destructive method using a Servo-Hydraulic universal testing machine (UTM) (ACE-USS200, Incheon, South Korea). For monitoring, the hydration temperature history of each concrete mixture specimen was measured using Smart Rock™ sensors. Each concrete mixture was tested for compressive strength using its cylindrical core, which was cured on days 1, 3, 7, 14, 21, and 28 after casting.

### 3.3. Structural Response and EMI

**Figure 9:**
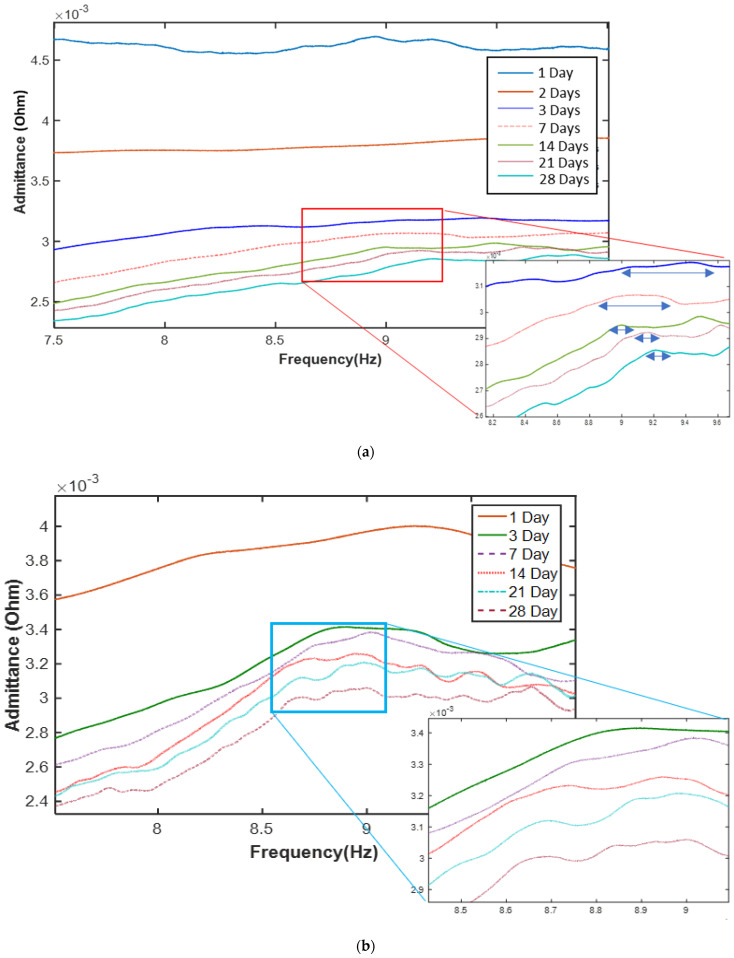
Electromechanical impedance (EMI) signature change with curing time of (**a**) concrete mixture type 1 and (**b**) concrete mixture type 2.

After casting the concrete in the formwork, the experimental setup was attached to the concrete specimens, and the data measurement process was started immediately. The responses of the signals propagating in the concrete paste depend on the hydration process. According to Mirmiran [51], the resonant frequencies of the signals in concrete are proportional to the stiffness of the material. Figure 9 presents the data of the impedance signature variation of mixture type 1 with time; specifically, a longer curing time is associated with a more smooth curve change.

The strength phase change in the concrete with the curing time is shown in Figure 9; it can be observed that the signatures change drastically with increasing curing age of the concrete. On the first day, the curve change is small and smooth; however, the curve shape is not constant because the concrete is in the initial sitting stage. An increase in the strength change is observed with the increasing curing time, from the 1st day to the 28th day of the curing period, for both concrete mixture types 1 and 2.

Regarding the features of the impedance data change, Giurgiutiu and Rogers conducted a promising study in which they used the EMI technique for structural health monitoring (SHM) in terms of damage detection. This procedure was also used by Kim. The impedance in the concrete media can be expressed as [52,53]

(7) $$Z_{conc}=i\omega m_{c}\left(\omega \right)\;+\;c_{c}\left(\omega \right)\;-\;\frac{ik_{c}\left(\omega \right)}{\omega }.$$

Here, $Z_{conc}$ is the is the electromechanical impedance of the host structure, $m_{c}$ is the mass, $c_{c}$ is the damping coefficient, $k_{c}$ is the static stiffness of the structure, and ω presents the excitation frequency.

Because the hybrid PZT–CNT sensors are embedded in the concrete specimens, the voltage applied by the sensors will generate vibrations in the concrete structures. Therefore, the dynamic change responses of the concrete specimens can be expressed as the mechanical impedance response of the PZT–CNT sensors. Thus, the data previously measured by the sensors are the effective EMI of the host structure. The sensor data can be expressed as follows:

(8) $$Z\left(\omega \right)\;=\frac{1}{i\omega C}{\left(1-\kappa_{31}^{2}\frac{\left.k_{str}\;(\omega \right)}{\left.k_{PzCn\;}+k_{str}(\omega \right)}\right)}^{-1}.$$

Here, $Z\left(\omega \right)$ is the EMI; C is the PZT–CNT zero load capacitance; $\kappa_{31}$ is the coefficient of the PZT ceramic cross-coupling for the EMI, which is specified in Table 1; $k_{str}\left(\omega \right)$ represents the specimen dynamic stiffness; and $k_{PzCn}$ is the PZT–CNT mean stiffness.

In this study, the EMI signature attenuation of the structures was investigated to predict the concrete strength with the change in the amplitude of the admittance. Using the EMI data and Equation (9), the 1-CC for the specimens were calculated. The 1-CC index further provides the information required for determining the dynamic change of the propagated signals.

(9) $$1-\mathrm{CC}=1-\frac{1}{N-1}\frac{\sum_{i=0}^{N}\left(Re\left(\bar{z_{0}}\right)-Re\left(\bar{z_{0}}\right)\right)\left(Re\left(z_{i}\right)-Re\left(\bar{z_{i}}\right)\right)}{az_{0}\;\sigma z_{i}}.$$

Here, *Re*(*z*_0_) is the impedance real part at the baseline and *Re*(*z_i_*) is the impedance real part at the *i*-th interval for each point of measurement.

**Figure 10:**
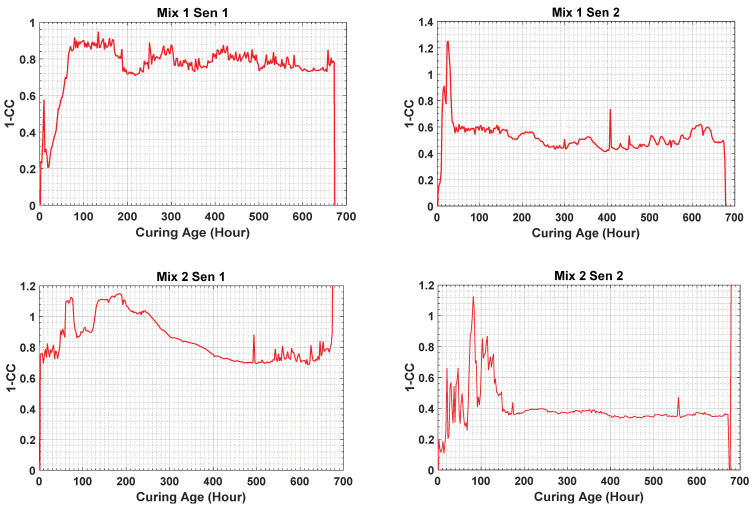
Cross-correlation data calculated for sensors 1 and 2 of both concrete mixtures.

Figure 10 depicts the 1-CC values measured from the EMI variation of the sensors with the curing time. The amplitude increase in the initial hours of curing occurs rapidly, which indicates a rapid phase variation of the material strength changing behavior. At the start of day 1, it increases rapidly and, at the completion of day 1, the amplitude rate of the change becomes lower.

### 3.4. Fuzzy Logic Tool

**Figure 11:**
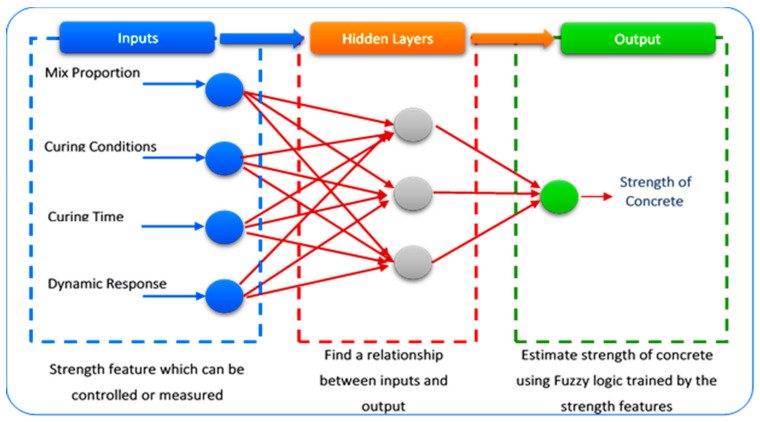
Fuzzy logic-based concrete strength forecasting model.

In this study, as the fuzzy logic tool has been used as the artificial intelligence (AI) data selection method, the model was prepared for obtaining noise free data from the data measured by sensors. As defined in recent studies of fuzzy logic theory, which relates various sets of objects having unsharp boundaries, for that selection, which depends upon the degree. For this study, keeping the result clear of high concentrated data, where the results vary with minor increase or decrease, we have implemented the fuzzy logic-based algorithm considering the perimeters of temperature, maturity, and curing age during the concrete curing period. The procedure of fuzzy logic was placed as depicted in Figure 11. For the data training for the features to calculate the concrete strength, in the implementation of fuzzy logic-based MATLAB model, several rules were fixed based on the features, for data screening process to give a lenient result for the calculation of 1-CC data. Applying block analyzing to get noise free data, we considered the EMI data for calculation of root mean square deviation (RMSD) and 1-CC for the clearing the parameters related to curing age and hydration temperature of the specimens.

## 4. Results and Discussion

### 4.1. Maturity Method Data

In the process of defining the maturity of the concrete and estimating the compressive strength, the hydration temperature history is used to predict the concrete strength using an NDT technique. It yields the features related to the hydration temperature history of concrete for calculating the concrete compressive strength. During construction using the concrete specimens, various techniques can be used to monitor the parameters of time and hydration temperature [54]. To dilute the concrete mixture, as water is added to the dry mix, chemical reactions commence between the aluminate, disilicate, trisilicate, and sometimes other admixture particles, forming a paste. The hydration temperature of the mixture increases in the early hours, i.e., during the initial sitting time and, subsequently, as the bonding in the concrete mixture strengthens, the hydration temperature reduces. N.J. Carino and other researchers have presented methods for calculating the maturity function from the temperature history [15,47]. As mentioned earlier, the cohesive bonding among the concrete particles is linear; therefore, the hydration temperature-resultant bonding shrinkage is a function of the curing age [15].

(10) $$S=S_{u}=\frac{\sqrt{k\left(t-t_{0}\right)}}{1+\sqrt{k\left(t-t_{0}\right)}}.$$

**Figure 12:**
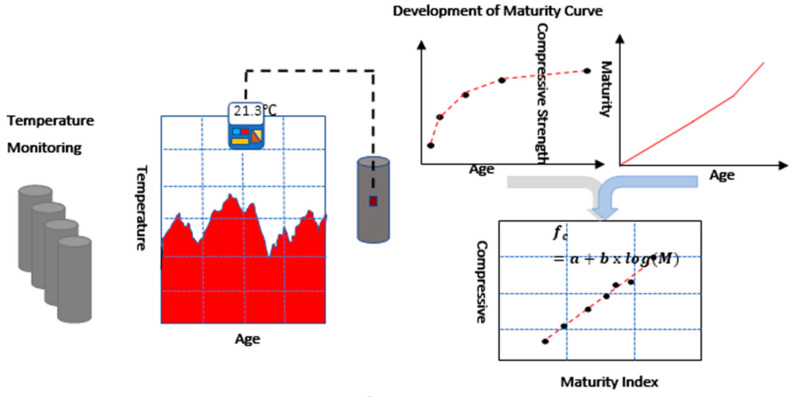
Process of monitoring the temperature, maturity index, and strength of concrete.

Here, the strength at time *t* is *S*; the initial sitting time is *t*_0_; and the limiting strength of concrete is $S_{u}$, which is the constant rate of energy transfer between the colliding molecules. As the system heats up, the kinetic energy of the molecules increases, and for energy lower reaction products, the molecules surmount the barrier. Thus, with increasing temperature, the reaction progresses at a constant rate of *k* [55]. The pictural procedure is presented in Figure 12.

The datum temperature value affects the value of the maturity index and, for different types of concrete, the datum temperatures are different, depending on the properties of the concrete type. According to the Nurse–Saul theory, the maturity can be expressed as

(11) $$M=\sum_{0}^{t}\left(T-T_{0}\right)\Delta t.$$

**Figure 13:**
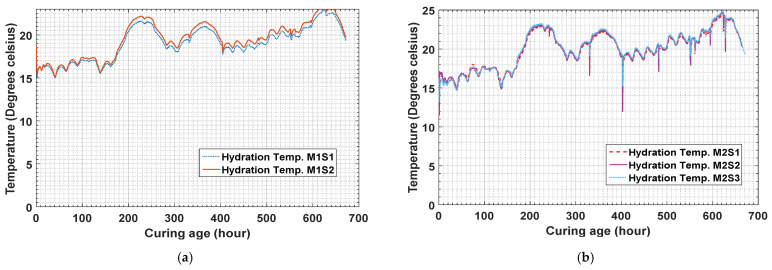
Hydration temperature history of embedded Smart Rock sensors (all) in concrete mixture types (**a**) 1 and (**b**) 2.

Here, *M* is the maturity function. This technique was applied experimentally, considering some parameters, to verify the strength data references. The temperature history of the hydration temperature shown in Figure 13 was measured by the sensors for specimens 1 and 2 of mixture type 1 and specimens 1, 2, and 3 of mixture type 2.

**Figure 14:**
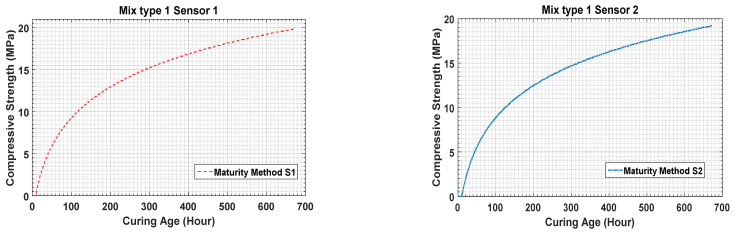
Maturity method strength result for concrete mixture type 1.

The temperature history reveals that the temperatures of concrete mixture type 1 are higher than those of concrete mixture type 2. This shows that the difference is a result of the concrete mixture type because concrete mixture type 1 has higher strength than concrete mixture type 2. Figure 14 shows that the strength gaining process starts after 1 h of the initial sitting. After the strength development in the early hours, the strength gaining process becomes rapid. Using the maturity method, the strength gained by concrete mixture type 1 is measured to be 2.98 MPa as by sensor 1 and 2.59 MPa by sensor 2 at the 24th hour after curing.

**Figure 15:**
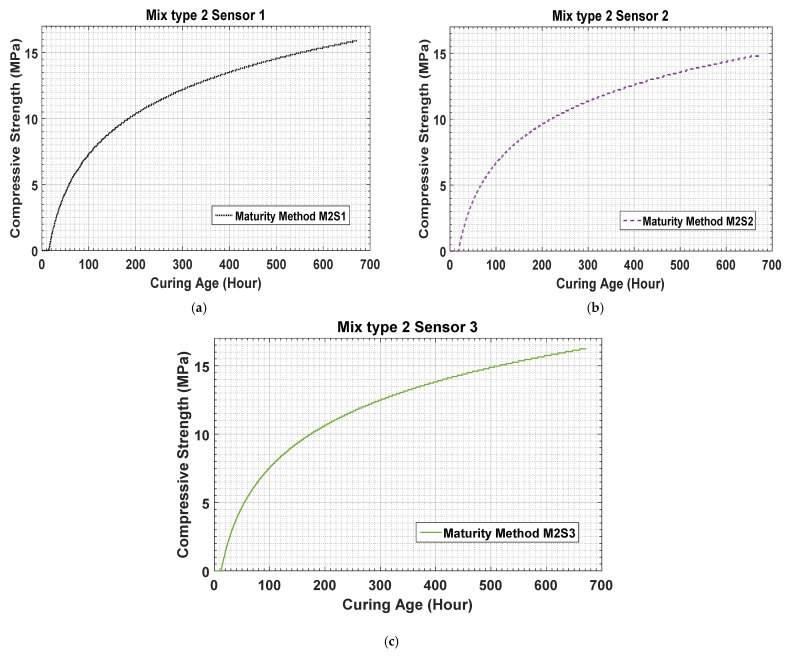
(**a**–**c**) Concrete strength data for concrete mixture type 2 obtained by the maturity method.

The results in Figure 15 present the data for concrete mixture type 2 measured by the temperature sensors. Sensors 1, 2, and 3 measured the strength as 1.69 MPa, 0.95 MPa, and 2.01 MPa, respectively. As the water–cement ratio of concrete mixture type 2 was higher, the process of concrete sitting started later than that for concrete mixture type 1, as shown in the figure.

**Figure 16:**
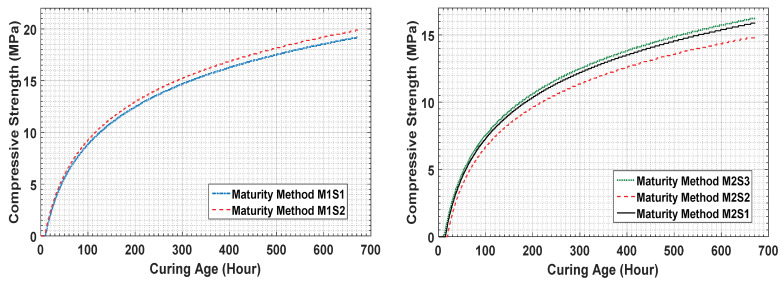
Comparative strength graph of concrete mixture types 1 and 2.

When the maturity method is used, the strengths of concrete mixture type 1 on the 28th day measured by sensors 1 and 2 are 19.88 MPa and 19.2 MPa, respectively. Similarly, the strengths of concrete mixture type 2 on the 28th day measured by sensors 1, 2, and 3 are 15.88 MPa, 14.78 MPa, and 16.22 MPa, respectively. The results for concrete mixture types 1 and 2 are compared in Figure 9 and Figure 16.

### 4.2. Hybrid PZT–CNT Sensor Data Results

The proposed model was applied to the data for the embedded sensors of concrete mixture types 1 and 2. Concrete mixture types 1 and 2 were monitored by two and three hybrid PZT–CNT sensors embedded in the specimens, respectively. Similarly, temperature sensors were also embedded in the concrete specimens to measure their hydration temperatures. The various parameters related to the concrete properties that were considered for estimating the concrete strength are listed in Table 3. These parameters were used with the algorithm of the fuzzy logic data screening method of probability selection. Before training the data, they were made noise-free for the 1-CC feature. Figure 16 depicts the modeling procedure for using the features of the concrete for strength forecasting.

**Figure 17:**
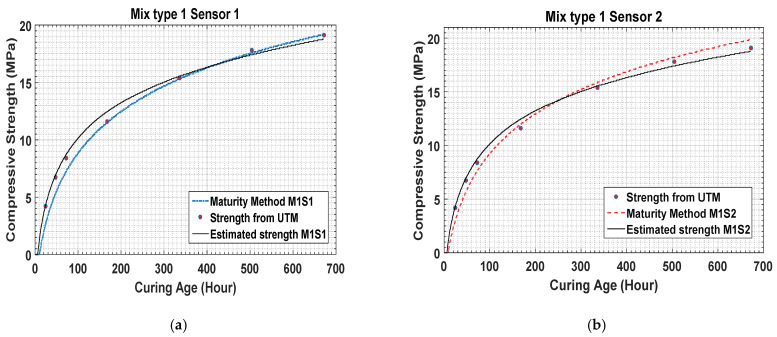
(**a**,**b**) Comparison of the estimated strength with maturity data and universal testing machine (UTM) results.

Following data acquisition from the sensors, strength estimation was conducted based on the maturity method procedure using the data measured by the temperature sensors. Figure 17a,b show the results for concrete mixture type 1 obtained using sensors 1 and 2. The cement–water ratio of this mixture was better; therefore, the change in the strength development curve started at an early age in the initial hours after casting the concrete. Initially, the strength gain was drastically high till the 48 h of the curing, following which the slope of the strength development curve presented a slower strength gaining process.

The results estimated for concrete mixture type 1 on day 14 revealed that, for specimens 1 and 2, the strength gains were 15.55 MPa and 16.02 MPa, respectively, which are approximately 80% of 28th day strength of concrete mixture type 1. This trend is observed because the hydration temperature of concrete mixture type 1 is higher than that of concrete mixture type 2.

**Figure 18:**
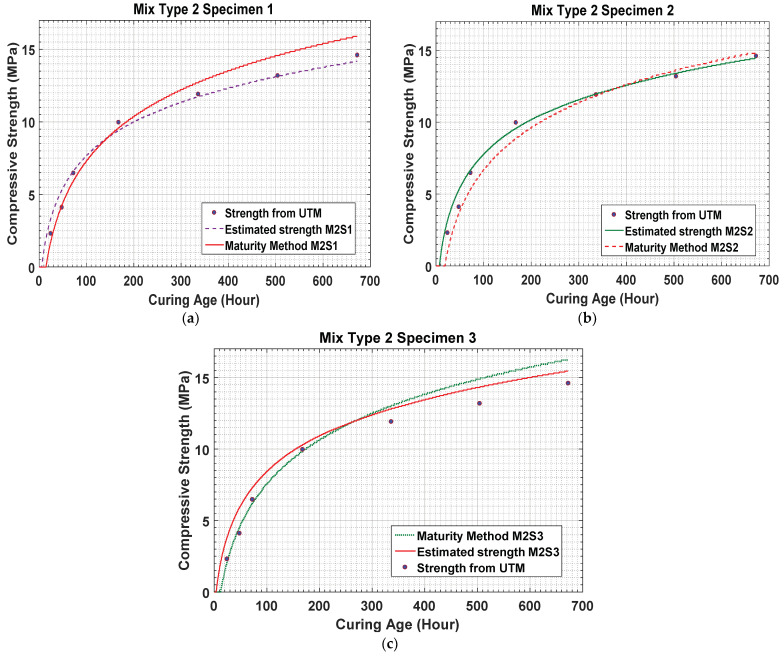
(**a**–**c**) Estimated data results for concrete mixture type 2.

For concrete mixture type 2, the results show lower strengths than those for concrete mixture type 1. Figure 18a–c compare the results obtained using sensors 1, 2, and 3 for concrete mixture type 2 and the UTM test results. For concrete mixture type 1, the predicted values are higher than those for the maturity method and UTM test results. Because concrete mixture type 2 contains an air entrainment agent, its actual strength establishment starts rapidly in the early hours of curing.

The figures show that, on day 1, the predicted strengths are 2.96 MPa, 3.25 MPa, and 3.8 MPa for sensors 1, 2, and 3, respectively. These values are higher than 2.3 MPa, which is the strength obtained by the UTM test. For concrete mixture type 2, the strength is gained rapidly in the earlier ages; in 72 h, it reaches 6.5 MPa. Figure 18a–c show that the strength establishment process continues rapidly till seven days after curing. Subsequently, the strength development curve changes gradually till the 14th day of concrete curing, following which it exhibits a more gradual development till the 28th day of curing.

**Figure 19:**
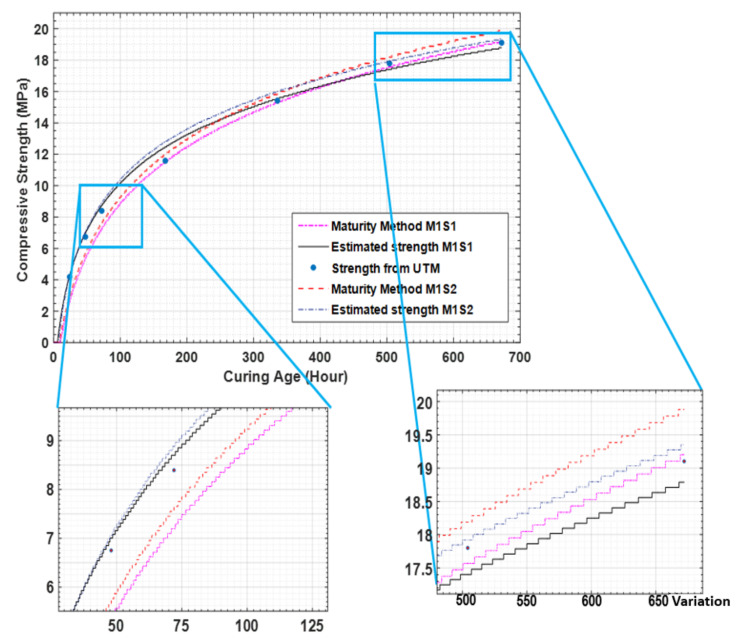
Estimated strength comparison with maturity data and UTM results for concrete mixture type 1.

For comparison, the estimated strengths of specimens 1 and 2 of concrete mixture type 1 are 18.78 MPa and 19.35 MPa, respectively, which are very similar to the strength gain obtained from the UTM test (19.1 MPa). Thus, the predicted results for concrete mixture type 1 are similar to its actual results. A comparison of the results for sensor 1 and 2 for concrete mixture type 1 is presented in Figure 19.

Table 4 summarizes the concrete compressive strength results obtained from the concrete cylindrical cores tested using the UTM. The values in the table are the mean values for different days after pouring the concrete.

**Figure 20:**
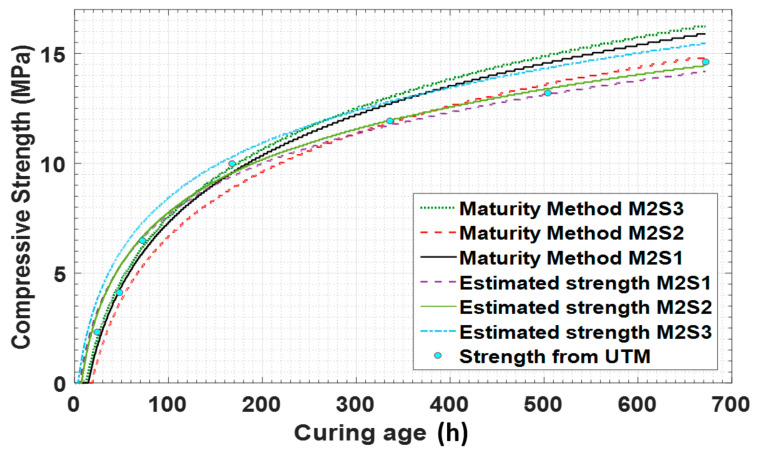
Maturity method, sensor data, and UTM test results for concrete mixture type 1.

Figure 20 compares the strengths obtained from the measured sensor data, maturity method, and UTM test for concrete mixture type 1. It can be observed that the results obtained using the three methods are similar, which demonstrates the potential of the proposed strength prediction technique, which is the comparison of concrete maturity method and EMI data for concrete strength.

**Figure 21:**
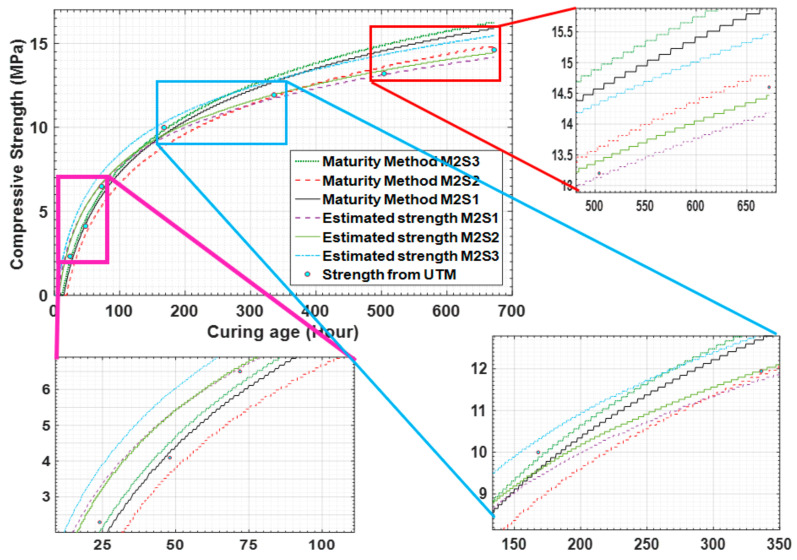
Estimated strength comparison with maturity data and UTM test results for concrete mixture type 2.

Figure 21 compares the maturity method, the fuzzy logic module-estimated, and UTM destructive testing results. It shows that, in the initial hours, the estimated results using the sensor data are relatively small in magnitude. This suggests that the data can be used in construction projects to ensure safe operation and prevent unwanted incidents.

On the 7th day, the estimated value by sensor 1 and the UTM result for concrete mixture type 2 are 9.54 MPa and 10 MPa, respectively. The strength estimation curve shows a similar pattern over the remaining days at strength pointed intervals. Therefore, Figure 21 clearly shows that this method can be used for the prediction of the compressive strength of fresh concrete.

## 5. Conclusions

The present study overcomes the limitations of concrete compressive strength development in the early curing days by analyzing and modifying various test methodologies for strength estimation. Smart materials, such as PZT material, CNTs, and some other alloys and materials, have excellent electromechanical properties; therefore, they can be used as sensors and actuators for different objectives in NDT.

The results of the study clarify the sensing effect enhancement of CNTs used as smart sensors by incorporating PZT materials, which exhibit excellent electrical and mechanical properties in a composite with cement and silica fume. In addition, at a high dispersion rate, the sensitivity of the CNTs increases, which clarifies their use as sensors.

From the results of the study, it can be concluded that the curing age time and the EMI signatures are the vital parameters in the measurement of structural dynamic responses, which can yield accurate prediction results for the designed concrete strength of a structure.

Regarding the maturity method, to calculate the maturity index, the datum temperature is important for each type of concrete and under every environmental condition. Therefore, it should be defined for each concrete mixture type. Here, we observed a rapid strength development at a high temperature.

The results of the study validate that the proposed technique can assist in SHM in massive industrial multi-step projects by expediting the process and ensuring structural safety. Moreover, the proposed method can be further integrated with other techniques, such as remote monitoring system, artificial intelligence technology, and so on, that may be structurally well-equipped for construction systems.

## Figures and Tables

**Table 1:** Properties of the attached piezoelectric (PZT) pitches.

WEB Series (APC Materials 850)		
Size of the PZT	Specimen Thickness	0.508 mm
	Dia	10.00 mm
PZT charge constant, 10^−12^ m/v	*d*_33_	400
	*d*_31_	−175
Electromechanical coupling factor	*k*_33_	0.72
	*k*_31_	0.36

**Table 2:** Details of concrete mixture types 1 and 2.

Specimen	W/C %	Unit Weight (kg/m^3^)						AD %	AE %
		W	C	GGBS ^1^	S. Fume ^2^	S	G		
1	40	175	328	228	0	717	723	0	0
2	34	185	330	0	0.165 (0.05%)	873	916	0.9	0.8

^1^ GGBS: ground granulated blast furnace slag. ^2^ S. Fume: silica fume.

**Table 3:** Parameters considered for concrete strength prediction.

	Sensor #	Parameters						Result
		Curing Age (h)	W/C	Environment Temp. (°C)	Hydration Temp. (°C)	1-CC	Maturity	Predicated Strength (MPa)
Mixture Type 1	S1 ^1^	24	35	24	16.42	0.275	2765.3	4.312
		72	35	24	16.55	0.83	46,572	8.701
		168	35	24	16.73	0.91	345,365	12.474
		336	35	24	19.71	0.79	586,347	15.554
		672	35	24	19.36	0.77	913,456	18.788
	S2	24	35	24	16.73	0.16	3634.7	4.124
		72	35	24	16.77	0.58	84,365	8.882
		168	35	24	17.03	0.53	416,692	12.768
		336	35	24	20.28	0.55	682,625	16.02
		672	35	24	19.75	0.499	847,562	19.351
Mixture Type 2	S1	24	31	24	15.89	0.8	4635.3	2.964
		72	31	24	17.91	0.61	63,594	6.64
		168	31	24	17.39	0.57	376,955	9.545
		336	31	24	21.29	0.549	703,254	11.976
		672	31	24	19.49	0.528	935,165	14.466
	S2	24	31	24	16.33	0.88	3352.5	3.253
		72	31	24	17.43	1.1	76,222	6.565
		168	31	24	17.47	1.12	383,462	9.412
		336	31	24	21.6	0.83	65,769	11.73
		672	31	24	19.1	0.82	872,399	14.176
	S3	24	31	24	15.93	0.38	5301.5	3.8
		72	31	24	17.56	0.69	72,954	7.283
		168	31	24	17.39	0.63	436,259	10.323
		336	31	24	21.78	0.71	685,294	12.793
		672	31	24	19.27	0.53	864,354	15.464

^1^ S1: These are different specimens used for concrete mixture type 1 and 2.

**Table 4:** Compressive strength results from concrete destructive tests.

Curing Age (Day)	1	2	3	7	14	21	28
Conc. Type 1 Strength (MPa)	4.2	6.75	8.6	11.6	14.4	17.8	19.1
Conc. Type 2 Strength (MPa)	2.3	4.1	6.5	10	11.94	13.2	14.6

## Data Availability

The data presented in this study are available on request from the corresponding author.

## Abbreviations

CNT	Carbon nanotube
EMI	Electromechanical impedance
NDT	Nondestructive testing
SWNTs	Single-walled nanotubes
UTM	Universal testing machine
MWNTs	Multi-walled nanotubes
APC	American piezoceramics
SHM	Structural health monitoring
PZT	Piezoelectric
CC	Cross-correlation
ASTM	American Society for Testing and Materials
